# Trauma-informed care in the UK: where are we? A qualitative study of health policies and professional perspectives

**DOI:** 10.1186/s12913-022-08461-w

**Published:** 2022-09-14

**Authors:** Elizabeth Emsley, Joshua Smith, David Martin, Natalia V. Lewis

**Affiliations:** 1 Centre for Academic Primary Care, Bristol Medical School, University of Bristol, Bristol, UK; 2 Centre for Academic Mental Health, Bristol Medical School, University of Bristol, Bristol, UK; 3 NIHR Bristol Biomedical Research Centre, University Hospitals Bristol and Weston NHS Foundation Trust and University of Bristol, Bristol, UK

**Keywords:** Psychological trauma, Mental health, Primary health care, Public health, Qualitative research, Policy making, Health policy

## Abstract

**Background:**

Trauma-informed (TI) approach is a framework for a system change intervention that transforms the organizational culture and practices to address the high prevalence and impact of trauma on patients and healthcare professionals, and prevents re-traumatization in healthcare services. Review of TI approaches in primary and community mental healthcare identified limited evidence for its effectiveness in the UK, however it is endorsed in various policies. This study aimed to investigate the UK-specific context through exploring how TI approaches are represented in health policies, and how they are understood and implemented by policy makers and healthcare professionals.

**Methods:**

A qualitative study comprising of a document analysis of UK health policies followed by semi-structured interviews with key informants with direct experience of developing and implementing TI approaches. We used the Ready Extract Analyse Distil (READ) approach to guide policy document review, and the framework method to analyse data.

**Results:**

We analysed 24 documents and interviewed 11 professionals from healthcare organizations and local authorities. TI approach was included in national, regional and local policies, however, there was no UK- or NHS-wide strategy or legislation, nor funding commitment. Although documents and interviews provided differing interpretations of TI care, they were aligned in describing the integration of TI principles at the system level, contextual tailoring to each organization, and addressing varied challenges within health systems. TI care in the UK has had piecemeal implementation, with a nation-wide strategy and leadership visible in Scotland and Wales and more disjointed implementation in England. Professionals wanted enhanced coordination between organizations and regions. We identified factors affecting implementation of TI approaches at the level of organization (leadership, service user involvement, organizational culture, resource allocation, competing priorities) and wider context (government support, funding). Professionals had conflicting views on the future of TI approaches, however all agreed that government backing is essential for implementing policies into practice.

**Conclusions:**

A coordinated, more centralized strategy and provision for TI healthcare, increased funding for evaluation, and education through professional networks about evidence-based TI health systems can contribute towards evidence-informed policies and implementation of TI approaches in the UK.

**Supplementary Information:**

The online version contains supplementary material available at 10.1186/s12913-022-08461-w.

## Background

Individual, interpersonal and collective trauma is a highly prevalent and costly public health problem [1]. The WHO World Mental Health Survey identified that 70% of participants had experienced lifetime traumas, including physical violence, intimate partner sexual violence, and trauma related to war [2]. People experiencing socio-economic disadvantage, women, minoritized ethnic groups, and the LGBTQ + community are disproportionally affected by violence and trauma [3, 4]. Adverse childhood experiences (ACEs) are stressful or traumatic events that occur during childhood or adolescence [5]. In England, a household survey found that nearly half of adults had experienced at least one ACE, including childhood sexual, physical or verbal abuse, as well as household domestic violence and abuse (DVA) [6]. DVA is considered to be a chronic and cumulative cause of complex trauma [7]. Up to 29% women and 13% men have experienced DVA in their lifetime, at a cost of £14 billion a year to the UK economy [7–9].

Cumulative trauma across the lifespan is associated with multiple health consequences [10]. The links between cumulative adversity from ACEs, DVA and other traumatic experiences are explained within the ecobiodevelopmental framework and the concept of toxic stress [11]. In a systematic review and meta-analysis of 37 observational studies of health behaviours and adult disease, patients with four or more ACEs were at higher risk of a range of poorer health outcomes including cardiovascular disease and mental ill health, versus those with no ACEs history [12]. Individuals and families who have experienced violence and trauma seek support from healthcare and other services for the physical, psychological and socioeconomic consequences of trauma [1, 13]. In the household survey in England and Wales, adults who had experienced four ACEs were twice as likely to attend their general practice repeatedly, compared with those with no ACEs history, and incidence of health service use rose as the ACEs experiences increased [14]. In a systematic review 47% of patients in mental health services had experienced physical abuse and 37% had experienced sexual abuse [15].

If the high prevalence and negative impacts of trauma are not recognised and addressed in healthcare services, there may be negative consequences for patients and healthcare professionals. Patients may not disclose trauma or recognise the impact of trauma on their health [16]. Patients may also be at risk of re-triggering and re-traumatization, for example by the removal of choice regarding treatment, judgemental responses following a disclosure of abuse, seclusion and restraint [17–19]. Re-traumatization within health services can affect both patients and members of staff, with the latter experiencing vicarious trauma [20]. The resulting chronic stress may impact on staff members’ ability to empathise and support others [21]. Many healthcare staff themselves have lived experience of trauma. A recent systematic review of healthcare professionals’ own experience of DVA, reported a pooled lifetime prevalence of 31.3% (95% CI [24.7%, 38.7%] [22].

Over last 20 years, several frameworks for a trauma-informed (TI) approach at the health systems level have been developed [13, 17, 23–28]. These frameworks aim to prevent re-traumatization in healthcare services and mitigate the high prevalence and negative effects of violence and trauma on patients and healthcare professionals. A TI approach (synonyms TI care, TI service system) starts from the assumption that every patient and healthcare professional could potentially have been affected by trauma [13]. By realising and recognising these experiences and their impacts, we can respond by providing services in a trauma-informed way to improve healthcare experience and outcomes for both patients and staff. The process of becoming a TI system is guided by key principles of safety, trust, peer support, collaboration, empowerment and cultural sensitivity [13]. The most cited frameworks for a system-level TI approach are those by Harris and Fallot [29], and the US Substance Abuse and Mental Health Services Administration (SAMHSA) [13]. These frameworks highlight that it is necessary to, firstly, change organizational culture and environments (organizational domain) and then change clinical practices (clinical domain) by incorporating the four TI assumptions and six TI principles throughout the ten implementation domains within a health system [13]***. ***Other authors proposed similar constructs for the framework of TI approach, often using slightly differing terminology [29, 30]. The authors consistently highlighted that the framework of TI approach is not a protocol but rather high-level guidance applicable to any human service system and should be tailored to the organizational and wider contexts. The process of becoming a TI system is described as a transformation journey rather than a one-off activity.

Despite a 20-year history of the TI approach framework, several reviews have found limited evidence for their effectiveness in health systems, with most studies conducted in North America and only one qualitative study in the UK [31–33]. Despite little evidence of acceptability, effectiveness, and cost effectiveness in the UK context, policies and guidelines at national, regional and organisational levels recommend implementing TI approaches in healthcare organisations and systems. It is important to understand how TI approaches are being introduced into policy documents, and how these policies are being interpreted and applied within UK healthcare. This study aims to understand the UK-specific context for implementing a TI approach in healthcare by exploring:

1. How are TI approaches represented in UK health policies?
2. How are TI approaches understood by policy makers and healthcare professionals?
3. How are TI approaches implemented in the UK?

This study of UK policy and practice will help us understand what TI approaches mean for policy makers and professionals to inform future UK-specific policy and TI approaches in healthcare.

## Methods

To answer our research questions, and consider perspectives from different standpoints, we conducted a multi-method qualitative study comprised of a document analysis of UK health policies followed by semi-structured interviews with key informants. Document analysis explored how TI approaches are represented in UK health policy while interviews explored professional views on how they are understood and implemented. We used the Ready Extract Analyse Distil (READ) approach [34], to guide the review of health policies and the framework method [35], to analyse data. The framework method is suitable for applied health research conducted by multi-disciplinary teams with varied experiences of qualitative analysis.

### Data collection

Data collection occurred between October 2020 and June 2021, with researchers and interviewees based in remote settings due to social distancing restrictions during the COVID-19 pandemic. Sample size was informed by the concept of information power [36], and restricted by the available funding and a tight timeline.

### Document search

We defined policy as ‘a statement of the government’s position, intent or action’ [37], and considered this definition at the level of a nation, local authority or organization. Two researchers (EE, NVL) identified key policy and related contextual documents, which provided background information on TI approaches. We identified documents through: (i) searches for peer reviewed and grey literature in our earlier systematic review on TI primary care and community mental healthcare [31], (ii) snowballing of references from included documents, (iii) signposting by interview participants and experts in the field of TI care. Researchers retrieved documents meeting the inclusion criteria: adult healthcare, UK-focus and discussion of TI approaches. We excluded documents on child healthcare, trauma-specific interventions and non-UK focus.

### Qualitative interviews

We conducted virtual semi-structured interviews with professionals at decision making levels who have direct experience of developing and implementing TI approaches in the UK healthcare system. We agreed to recruit up to 10 professionals from national and local governments and healthcare organisations. Researchers sent an expression of interest letter via email and Twitter to: (i) individuals and professional networks of policy makers, (ii) authors of included policy documents, (iii) individuals recommended by interview participants. Interested individuals contacted study researchers who checked their eligibility, sent participant information leaflets, answered questions, and arranged interviews with those eligible and willing to proceed. Interviews were conducted over the Zoom video call platform. Researchers obtained verbal informed consent, asked demographic questions, and followed a flexible topic guide to ensure primary issues were covered during all interviews but allowing participants to introduce unanticipated issues. The topic guide explored participant experiences of developing and implementing TI approaches and their views on how TI approaches have come to be represented in policy and implementation (Additional file 1). The interviews were audio-recorded with consent, professionally transcribed verbatim, and anonymised.

### Analysis

Data analysis started alongside data collection, to help refine and guide further data collection [35]. We followed the four-step READ approach to document review in health policy research: 1) ‘Ready your materials’ which involves agreeing the type and quantity of documents to analyse, 2) ‘Extract data’ whereby key document information such as basic data and concepts are organized, 3)’Analyse data’ when data is interpreted and findings are developed, 4) ‘Distil your findings’ which involves assessing whether there is sufficient data to answer the research question and findings are refined into a narrative [34].

In step one, two researchers (EE, NVL) agreed to use purposive sampling to gather 24 documents representing a broad range of document categories including primary legislation, parliamentary documents, NHS and Public Health England strategy and planning documents, service-user perspectives, evaluation reports, and guidance on ‘how to do’ TI approach [38]. EE ordered included documents chronologically. In step two, EE read and re-read all included documents and used a customized Excel form to extract data on document title, authors, year, source, objectives, target audience, focus, key messages, referenced evidence, policies/guidelines, and recommendations. During data extraction, researchers made notes about how each document answered the following questions: What is TI care? TI care for whom? Why TI care? EE and NVL met regularly to discuss preliminary ideas for analysis.

In step three, we imported all included documents and interview transcripts into NVivo R project and applied the framework method [35]. To address variability in definitions and terminology regarding TI approaches, we included key concepts from the well-known SAMHSA system-level framework [13], as a basis for our coding frame, for example the six TI principles. First, all researchers read four documents and two interview transcripts and independently manually coded text relevant to our research questions using a combination of inductive and deductive coding [39]. Deductive coding helped to identify concepts related to TI care, even if the document itself did not specifically use the “trauma-informed” term. The researchers then met to compare initial thematic codes and agree on a ‘working analytical framework’ which was imported into NVivo and applied to the documents and interviews transcripts. We refined the framework by adding and merging thematic codes identified subsequently, ran matrix coding queries by data sub-sets (documents, interviews), and combined codes into final analytical themes that answered our research questions. During the analysis stage, researchers met bi-weekly to finalise the dataset, develop and refine coding frame and themes. We wrote reflective diaries and analytical notes and discussed how our clinical backgrounds in general practice and psychiatry, and varied experiences of qualitative research, could have influenced the analysis.

In step four, we stopped document review when we reached the pre-specified number of documents and discussed common findings. First, we illustrated how TI approaches have developed in the UK over time by creating an integrated timeline with document publication dates, the years when interview participants began working in this area, and broader contextual factors from national news and related media. Then we integrated findings from the analysis of documents and interviews through three iterative cycles of developing final analytical themes cutting across documents and interviews. Researchers produced written accounts of the themes, and tables with illustrative quotes that explained how TI approaches have been represented in policy documents, understood, and implemented in the UK.

## Results

### Policy documents

We identified 50 documents and selected 24 policy documents at national, local, and organizational levels. The remaining 26 documents provided context and a background on TI approaches. The documents included were published over nine years (2012–2021) and considered all UK nations, multiple sectors, government policy and service-user voices. The documents either mentioned a TI approach or discussed related concepts such as a patient choice and safety of services (Table 1).

**Table 1:** UK policy documents included in analysis, ordered by publication date and category

Document category	Document year and title
**Primary legislation, white papers, and consultation reports**	2012 Health and Social Care Act [40] 2019 Transforming the response to domestic abuse: consultation response and draft bill [41] 2021 Integration and Innovation: working together to improve health and social care for all [42]
**Government and parliamentary documents**	2016 Ending violence against women and girls. Strategy 2016–2020 [43] 2018 Public Health Priorities for Scotland [44] 2018 HM Government: Serious Violence Strategy [45] 2019 Ending violence against women and girls 2016–2020: refresh strategy [46] 2021 COVID-19 Mental Health and Wellbeing Recovery Action Plan [47]
**NHS/Public Health England strategy documents**	2014 Five year forward view [48] 2018 Strategic direction for sexual assault and abuse services – Lifelong care for victims and survivors: 2018–2021 [49]
**NHS/Public Health England planning documents**	2019 NHS Long Term Plan [50] 2019 NHS Mental Health Implementation Plan 2019/20–2023/24 [51]
**Patient/service-users and carer perspectives**	2019 Engaging with complexity: providing effective trauma-informed care for women [52]
**Evaluations and evidence reported to Department of Health or local government**	2018 The Women’s Mental Health Taskforce. Final report [17] 2019 A sense of safety: trauma-informed approaches for women [53] 2019 Developing trauma informed practice in Northern Ireland: key messages [54] Successes and struggles keeping trauma in mind: development of a trauma informed adult mental health service [55] Ten evidence-based reasons for embedding values-based ‘Enabling Environments’ in health care [56]
**Guidance on ‘how to do’ trauma-informed care’**	2017 Transforming psychological trauma: a knowledge and skills framework for the Scottish Workforce [25] 2018 Trauma-informed approaches. Trauma-informed care: implications for practice [57] 2018 Trauma-informed care in response to adverse childhood experiences [58] 2018 Adverse Childhood Experiences, Resilience and Trauma-informed care: a Public Health Approach to Understanding and Responding to Adversity. The Annual Report of the Director of Public Health [59] 2019 Developing real world system capability in trauma informed care: learning from good practice [60] 2021 A good practice guide to support implementation of trauma-informed care in the perinatal period [28]

*Note*: *NHS* National Health Service

Mirroring the historical development of TI approaches from mental health services [13, 29], across both documents and interviews, mental health was the most referenced sector (*n* = 24), followed by women’s health (*n* = 11), healthcare for rough-sleepers (*n* = 7), primary care (*n* = 4) and major incident management (*n* = 1). The level of application of the TI approach varied from one organization [55], to a public health board [59], to NHS-wide [48, 50]. The geographic coverage of policy documents ranged from UK wide (*n* = 10) to regional application (*n* = 24). Scotland emerged as a leading region with the TI knowledge and skills framework for the Scottish Workforce [25].

**Fig. 1:**
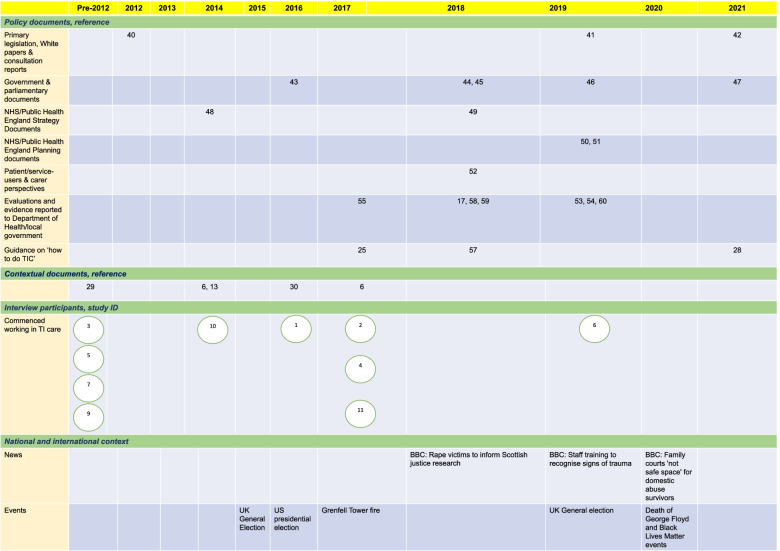
An integrated timeline of how TI approaches have developed in the UK. Document publication dates, the years when interview participants began working in this area, and broader contextual factors from national news and related media are captured. The number/s in each cell correspond to a document reference [6, 13, 17, 25, 28–30, 40–55, 57–60]

The timeline of TI approaches and related concepts in the UK showed a steady growth between 2012 and 2021 with parallel developments from top-down and bottom-up (Fig. 1).

We identified few documents prior to 2012, with the Health and Social Care Act published in 2012. Although the Act did not specifically use the term TI care, it discussed related concepts of a greater voice for patients, enabling patient choice and safety of services. We found a noticeable clustering of documents in 2018 and 2019. Potential contributions could be the release of key contextual documents such as the US SAMHSA guidance and the National ACEs Study in the preceding years [13, 14]. Other possible reasons could be the high-profile MeToo and Black Lives Matter movements and tragedies like Grenfell fire. Relevant news articles, including calls for rape victim support and professional training on trauma, came to the fore in 2018–2021. These events and activities have brought the issues of trauma, vulnerable populations, intersectionality, and racial justice to the foreground and may have helped achieve a focus on TI approaches as a responsive system-level framework.

### Qualitative interviews

In total, 21 professionals expressed interest, 2 did not have direct experience of TI approach at the system level, 8 did not respond by the deadline, 11 provided consent and were interviewed. Interviews lasted between 32 and 68 min (mean 52 min). We achieved a maximum variation sample representing diversity of gender (4 men, 7 women), organizations (public, private, third sector), professional role (frontline to leadership positions), and direct experience of developing and/or implementing TI approaches in healthcare (from 2 to 25 years). Most participants developed and implemented TI approaches in England, at the level of organizations and local authorities (Table 2).

**Table 2:** Characteristics of interview participants

Participant ID	Gender	Geographical area	Organization	Current role	Role in developing and/or implementing trauma-informed approach	Years of direct experience
**1**	Male	Wales Northwest England	Local authority Charity	Freelance public health practitioner	Training, consultancy	5
**2**	Male	East Midlands, England	NHS Mental Health Trust	Clinical lead for trauma service	Research, clinical work, training	4
**3**	Female	North England	NHS Mental Health Trust	Clinical director of a service	Led implementation	21
**4**	Female	Southeast England UK-wide	NHS Mental Health Trust National charity	Women's services lead Trainer, consultant	Chose and managed implementation, training	4
**5**	Female	Southwest England	Charity for people with complex needs and substance misuse	Senior manager	Wrote organization's policy, chose and managed implementation, training	17
**6**	Female	Southwest England	Mental health and housing charity	Senior psychologist	Led the design and implementation of trauma-informed approach in the organization	2
**7**	Male	Northwest England	Private consultancy	Company director, consultant clinical psychologist, visiting professor	Designed and delivered training, supported organizations to develop trauma-informed approach	25
**8**	Female	UK-wide	UK body of the international charity delivering mental health and support services	Senior psychosocial practitioner	Developed organization's trauma-informed approach, developed national policy, produced educational material for a national body	-
**9**	Male	Southwest England	NHS Mental Health Trust	Trauma-informed care lead	Developed trauma-informed approach for mental health trust, led implementation	10
**10**	Female	Northwest England	Charity	Trauma-informed care lead	Research, implemented town-wide trauma-informed approach across various services, member of international network on trauma-informed care	7
**11**	Female	East Midlands, England	Local authority	Public health manager	Managed commissioning of training for several groups of healthcare professionals within local authority	4

*Note*. *NHS* National Health Service

Three out of ten interview participants had been involved in developing and implementing TI approaches prior to the release of the first document in 2012, with the rest becoming involved in 2017, just prior to the clustering of documents in 2018 and 2019 indicating a pivotal wave of popularity of the TI approach framework at this time. Participants explained that their clinical practice facilitated interest in the topic.

Our framework analysis has produced three analytical themes with seven sub-themes:

1. How TI approaches are represented in UK health policies
2. How TI approaches are understood
  TI care as different from other practices
  TI care as a contextually tailored organizational approach
  TI care as a remedy to challenges;
3. How TI approaches are implemented
  Piecemeal implementation and a need for a shared vision
  Factors that facilitated or hindered implementation
  The evidence-policy gap
  The future of TI care in the UK

### How TI approaches are represented in UK health policies

We found that the TI approach is referenced in government initiatives and included in policies at a national level, as well as in NHS and non-NHS organizations, local authorities, and devolved nations; however, there was no dedicated strategy or a position statement, nor was there an agreed terminology and framework, or a robust evidence base in the UK. Despite growing endorsement of TI approaches in policy documents (Fig. 1), positive statements at the national and NHS level were not backed up with legislation, guidance, funding commitment, and resource allocation.

### How TI approaches are understood

#### TI care as different from other practices

We found divergent interpretations of a TI approach versus other concepts related to trauma, such as ACEs, psychologically informed environments and standard good clinical practice. One participant unified concepts such as TI care, ACEs and psychologically informed environments in recognising past traumatic experiences. Another participant detached the terms ACEs and TI care, reflecting that ACEs have become well known in research whereas a TI approach is a pragmatic way of supporting those who have experienced trauma. All documents and most participants clearly differentiated between a TI approach at the system level and standalone TI practices (e.g., routine enquiry about ACEs, one-off training about trauma). However, some participants considered standalone TI practices to be a TI approach. Documents and most interviewees differentiated TI approach from a good clinical practice by incorporation of the TI assumptions and principles [25].

#### TI care as a contextually tailored organizational approach

In line with the SAMHSA guidance [13], document and interview data showed that the framework of a TI approach needs to be tailored to the organizational and wider context. Policy documents advised organizations to clarify what TI care means for them, and that application of the framework should depend on the needs of service users and organisations [25, 28, 52, 54, 59, 60]. Several documents suggested that this organizational tailoring should be informed by service-users through co-production and co-design of services [17, 28, 49, 52–55, 60, 61].

#### TI care as a remedy to challenges

In all policy documents and in nine interviews, TI approaches were presented as a remedy to a variety of problems within health systems. Sixteen of twenty-five documents justified a TI approach as a way for addressing the high prevalence and negative impact of violence and trauma on patients, with eleven documents considering its impact on staff. The growing international evidence base for the impact of psychological trauma and the need for service response was used in documents and interviews to justify TI approaches as a pragmatic solution to these concerns. However, the documents and interview participants justified the need for TI care by citing US and Welsh epidemiological studies on ACEs, DVA and patient accounts of being re-traumatized in services. We found no references to intervention studies that demonstrated effectiveness, cost-effectiveness, or acceptability of TI approaches in the UK.

In the NHS Long Term Plan, TI care was also identified as a component of a new model of integrated care [50]. A TI approach has also been presented as a solution to addressing the collective trauma of the COVID-19 pandemic for patients and staff [62].

### How TI approaches are implemented

#### Piecemeal implementation and a need for a shared vision

Interviewees confirmed the piecemeal implementation of TI approaches in the UK and felt that a shared national vision would be beneficial. Participants agreed that the implementation of TI approaches varied across the UK, with Scotland having more strategic coordinated implementation (additional file 2, quote 1). We found that different regions and organizations reinvented the TI approach wheel, with interviewees expressing a need for national coordination. Participants expressed the need for adequate allocated resources and a more unified approach across organizations and sectors as a solution to the patchy implementation in England (additional file 2, quote 2). They gave examples of the bottom-up networking initiatives driven by experts in TI care who created opportunities for sharing best practice and resources for implementing TI approaches. Participants cited a UK-wide Trauma Informed Community of Action and local TI care working groups.

One participant from England suggested that whilst the SAMSHA definition of TI approach was widely cited, they did not feel there was an agreed set of components and activities for implementing the framework in practice. This participant felt that a consensus on shared practice standards was a necessary next step for TI care in the UK.

#### Factors that facilitated or hindered implementation

At the organization level, some participants felt high level leadership support was needed, and if lacking is a barrier to implementing a TI approach. To achieve effective implementation leaders with power and those with passion were felt to be important. The concept of organizational champions garnered support when “*champions act as influencers and their credibility within services adds to the potential for buy-in from other staff*”, fostering sustainable change [53]. One participant warned against a reliance on top-down leadership, explaining that when a senior leader leaves an organization’s priorities can change. The participant also felt that change driven from the top-down, might lead to resistance from front-line staff (additional file 2, quote 3). Some interviewees reaffirmed the view that people with lived experience should be involved in leading implementation of TI approaches (additional file 2, quote 4).

Some interviewees felt that passionate individuals alone cannot create effective change without support at the organization level (additional file 2, quote 5). Collective responsibility and organizational commitment were highlighted as an essential factor to support individuals with passion. In contrast, unsupportive organizational culture and high-pressure environments was perceived as a barrier (additional file 2, quote 6). One document cited scarcity of resources and low staff morale, as well as a resistance to new initiatives and upheaval [52]. Competing demands and opportunity costs were also raised (additional file 2, quote 7).

At the wider context level, documents highlighted the value of political support capable of influencing practice nationally [17]. Some interviewees explained disconnected and decentralized implementation of TI approaches across the UK by a shortage of political will and leadership in the central UK government, compared with those of the devolved administrations in Scotland and Wales (additional file 2, quote 8). Proposed explanations included smaller territories, populations and governments, and “*more of a left-leaning social conscience politics” (Participant 3).* Another interviewee called for a united parliamentary leadership recognised by government and capable of influencing policy.

Inadequate funding and commissioning of services was also described as a barrier, partly explaining regional differences in implementation of TI care (additional file 2, quote 9). The COVID-19 pandemic was perceived as a barrier that contributed to the backlog of initiatives and work in the pipeline (additional file 2, quote 10).

#### The evidence-policy gap

UK policies on implementation of TI approaches were not supported by UK-specific, methodologically robust, evidence for effectiveness, cost effectiveness and acceptability. Participants explained the policy-evidence gap by citing methodological challenges of evaluating system-level transformation and a need for commitment from commissioners and funders (additional file 2, quote 11). In addition, participants who developed and implemented TI approaches in their organizations and regions did not have the capacity to evaluate their initiatives and disseminate the findings (additional file 2, quote 12).

#### The future of TI care in the UK

Participants had differing views on the future of TI care in the UK, although most agreed on its permanency. Some interviewees felt that TI approaches have already gained a critical momentum in the UK. In contrast to comments about TI care as a passing trend or ‘buzzword’ in the absence of in depth understanding, several interviewees voiced confidence that TI approach is here to stay, and will evolve, being incorporated into policy as well as being adopted more widely. Others were less optimistic and were concerned that insufficient political backing means policy endorsement will not translate to meaningful practice change.

Some participants thought that TI care should become a mandatory consideration with stronger central policy or monitoring by national watchdogs. They felt that the support of additional nation-wide regulatory measures could be beneficial. In contrast, some interviewees showed scepticism, fearing the creation of further ‘box-ticking’ measures. They feared that efforts to police or monitor providers could create a burden of empty bureaucracy without improving practice (additional file 2, quote 13).

## Discussion

Our document analysis of health policies and interviews with professionals found differing representation, understanding, and implementation of TI approaches in the UK with wide variations between geographical areas, services, and individual professionals. Cross-sectoral endorsement of TI approaches in policies was not supported by high-level legislation or funding, and a UK-specific evidence base. Despite divergent and conflicting interpretations of TI approaches, the common understanding was that it differs from other practices by integrating TI principles at the organisational level and it should be tailored to the organization and wider contexts. It can also address NHS problems from integrated care to post-COVID recovery. We found more centralized implementation of TI approaches in Scotland and Wales versus piecemeal implementation in England. The implementation of TI approaches in England was driven from the bottom-up by passionate dedicated leaders at the level of organization or local authority, who called for more coordinated working supported by the UK government and NHS leaders. We identified factors that facilitated or hindered implementation of TI approaches at the level of organization (leadership, service user involvement, organizational culture, resource allocation, competing priorities) and wider context (government support, funding). The evidence-policy gap in TI care implementation can be explained by limited funding and evaluation capacity. Professionals had differing views on the future of TI approaches, however all agreed that without political backing at the government level, policy endorsement will not translate into meaningful implementation.

Our finding of a marked difference in the landscape of TI approaches in healthcare systems between the devolved nations, with evidence of a unified national strategy emerging in Scotland and Wales and notably absent in England could have several explanations. These include smaller territories, populations, and governments in devolved nations, with clear buy-in from government-level leadership in Scotland. Our analysis highlighted the initiatives of local decision-makers in England who have developed and implemented TI approaches in their own organizations and local authorities. The absence of a national strategy in England contributed to the piecemeal implementation, with some regions leading the way, and others silent. As local TI leads have been left to ‘find their own way’, they may not always have been aware of similar initiatives in other organizations and regions. A proposed solution was bottom-up initiatives aiming to bring the local TI leads together to share resources and good practice. This finding indicates the need for a leader on TI approaches within or linked to the UK government who can support and strengthen the bottom-up initiatives.

Another important finding is confirmation of the evidence-policy gap, with proposed reasons emerging in the analysis. Interview participants explained an absence of UK evidence on the effectiveness of TI approaches by a need for more interest from commissioners and funders, as well as a lack of physical and methodological capacity to evaluate system-level TI approaches. The former can be resolved through funding calls and comprehensive, transparent evaluation. The latter can be addressed by funding evaluations and raising awareness regarding available methodologies and tools for evaluating TI system change interventions [32, 33].

Our finding of differing understanding of TI-approaches is in line with prior literature [63]. We found that some participants interpreted standalone TI practices (e.g., ACEs enquiry, one-off training about TI care) as a TI approach. Such interpretations are not supported by evidence. Authors of the ACEs study explained that the ACEs score is not a diagnostic tool, therefore care should be taken if used as part of community-wide screening, with rigorous evaluation of its use [64]. Recent reviews also found limited evidence on outcomes from routine enquiry, recommending further research [65, 66]. Several systematic reviews demonstrated that standalone awareness raising did not result in change in behaviour and practices among healthcare professionals [67, 68].

These misunderstandings can be explained by the conceptual mutability of a TI approach framework, lack of awareness about existing frameworks, and a need for coordinated working led by experts in TI approaches. The evidence of emerging working groups and UK-wide professional networks on TI care is promising. However, these initiatives require adequate funding and coordination to sustain momentum and develop further. These professional networks can become the platform for education about evidence-based TI approaches contributing to increasing value and reducing waste in research and implementation in this field.

This study is methodologically robust with perspectives drawn from UK policy documents and professionals, who have direct experience of developing and implementing TI approaches. Data analysis occurred alongside data collection, to help refine and guide further data collection. The limitations include no professional informants from devolved nations and no participants at the level of UK government. Due to time and funding restrictions, we could only recruit 11 professionals and did not interview patients including those with lived experience of trauma. Our small sample size could have resulted in underrepresentation of views of some stakeholders. Future research should recruit informants from these groups to draw a complete picture of the landscape of TI approaches in the UK.

## Conclusions

Although health policies endorse implementation of TI approaches in the UK, they do not provide specific legislation, strategy or funding and are not supported by evidence of effectiveness. Understanding and implementation of TI approaches varies between regions, organizations, and individual professionals; however, all agree that if implemented at the system level and contextually tailored, TI approaches can mitigate varied problems withing NHS. The implementation of TI approaches in the UK is driven by local experts in TI care. A coordinated, more centralized strategy and enhanced provisioning for TI healthcare, including increased funding for evaluation and education through TI professional networks, can contribute towards evidence-informed policies and implementation of TI approaches in the UK.

## Supplementary Information

**Additional file 1. **Interview topic guide.

**Additional file 2. **Analytical themes with subthemes and supporting quotes.

## Data Availability

Data are available at the University of Bristol data repository, data.bris, at https://doi.org/10.5523/bris.2awc5pqkavac12d6jm1qp9wetm. For reference: Lewis, N. (2022): TAPCARE policy review study. https://doi.org/10.5523/bris.2awc5pqkavac12d6jm1qp9wetm All methods were performed in accordance with relevant guidelines and regulations as detailed here: https://www.biomedcentral.com/getpublished/editorial-policies#research+involving+human+embryos%2C+gametes%2C+and+stem+cells.

## Abbreviations

ACEs: Adverse childhood experiences
COVID: Coronavirus disease
DVA: Domestic violence and abuse
NHS: National Health Service
SAMHSA: Substance Abuse and Mental Health Services Administration
TI: Trauma-informed
UK: United Kingdom
US: United States of America
