# Supplementary material for: Trauma-informed care in the UK: where are we? A qualitative study of health policies and professional perspectives
Source: BMC Health Serv Res. 2022 Sep 14;22:1164. doi: 10.1186/s12913-022-08461-w (PMC9473455; doi:10.1186/s12913-022-08461-w)
Supplement: Supplementary file 1 — Additional file 1. Interview topic guide. [file 12913_2022_8461_MOESM1_ESM.docx]

| 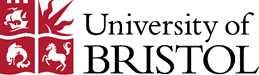 |  |
| --- | --- |

# TAP CARE study: analysis of policy and professional perspectives on trauma-informed primary and community mental healthcare

## Interview topic guide

Welcome participant and thank them for attending. Introduce yourself (if multiple roles, introduce all).

**Part A: Introduction and verbal consent**

- Follow Verbal consent interview script v3 19.03.21 and record as a separate file

**START RECORDING INTERVIEW**

**Part B. Establishing rapport:**

- Find out why they felt they wanted to take part in the research.

**Part C: Demographic information**

- Job title, type of organisation
- Describe current organisation, its role in TIC health care.

**Part D: Main body of interview**

1. Direct experience of work on TIC in health care:

- What TIC work did you do?
- In what capacity?
- Length of time involved in TIC?
- What was it like?
- Achievements.
- Challenges.

Prompts for barriers: lack of resources, disjoint approach across sectors, politically driven.

- How they addressed these challenges?
- How were their TI interventions evaluated?

1. Explore what they know about policies/guidance (high level, local) on TIC, what they say, how they work in practice.
2. When reviewing research evidence and policies on TIA to healthcare, we found that there is a gap. National and local policies already endorse TIA despite little evidence of their effectiveness, acceptability, and cost effectiveness in the UK context.

- Why do you think the concept of TIA has appeared in national and local UK health policies?

Prompts: political, economic, moral drivers; USA influence.

- What are the reasons for the gap between research evidence and policies?
- How we can address this gap?

1. In reviewing literature of trauma informed care, the term ACEs comes up a lot, especially in the UK context. There seems to be variation in what extent policy makers consider ACEs when writing about trauma informed care.
   - Why do you think this is?
   - To what extent should trauma informed care incorporate an understanding of ACEs
   - Do you think ACEs and trauma informed care should be included in policies together or separately?
   - Is the concept of ACEs important to trauma informed care?
2. Explore their view on the UK variations in how TIA to care is represented in policies.

We found that England, Scotland, Wales, and Northern Ireland took different approaches to TIA to healthcare (e.g., a system -wide approach in Scotland and Wales, patchy in England).

- What are the reasons for such variation?

1. Explain that some GPs and researchers asked us about the difference between TIC and the good quality usual care they already provide. Can you explain the difference to me?
2. What are your thoughts on the effect of the coronavirus pandemic on trauma informed approaches to healthcare?
3. Explore their views on the future role for TIA.

- What is your view on the future of TIA to healthcare nationally/locally?

**Part E: Closure of interview**

- Explain and briefly look through question sheet to check that you have covered all the questions.
- Let the participant know that that is the end of the questions and ask them if there is anything else they feel they would like to say or add?
- Ask what job title and work place they want you to use with their quotes in publications.
- Ask the participant if there were any questions that were confusing or difficult to answer? (If so, which and in what ways?)
- Thank them for taking part.

**STOP RECORDING INTERVIEW**

Follow ‘TAP CARE interview SOP v2 2021.03.19’ section **After interview**
