# Supplementary material for: Trauma-informed care in the UK: where are we? A qualitative study of health policies and professional perspectives
Source: BMC Health Serv Res. 2022 Sep 14;22:1164. doi: 10.1186/s12913-022-08461-w (PMC9473455; doi:10.1186/s12913-022-08461-w)
Supplement: Supplementary file 2 — Additional file 2. Analytical themes with subthemes and supporting quotes. [file 12913_2022_8461_MOESM2_ESM.docx]

Additional file 2. Analytical themes with subthemes and supporting quotes

| **Subthemes** | **Supporting quotes** |
| --- | --- |
| How TI approaches are implemented | |
| Piecemeal implementation and a need for a shared vision | Quote 1  “They [Scotland] have a national programme for this that cuts across all of the services… They’re looking at it much more systemically and making sure that the training and the learning is happening systemically. In England, at best, it’s piecemeal, it’s seen as relevant for that group but not this group. I hear very little conversation about it in relation to systems. I hear very little real depth of understanding.” Participant 3  Quote 2  “I couldn’t agree more that it is so patchy across England. And actually, it makes work very challenging because you are doing something, and then all of a sudden you find out that somebody else in the south is doing the same piece of work. But actually, you could have been working together on it if the resources were there and you could all have- If there was a hub for us to be able to do this work, then things would be a hell of a lot easier.” Participant 10 |
| Factors that facilitated or hindered implementation | Quote 3  “I think change is best when it comes from the bottom up. Where we can create a movement of people who want to be trauma-informed…The more top-down things are, the more likely you are to get resistance.” Participant 9  Quote 4  “I really hope that people who have experienced some of the challenging experiences, that as professionals we might talk about, are the leaders in that…Professionals can step back and support and steward, if you like, but really hold in mind these power dynamics within it.” Participant 6  Quote 5  “The reason I think it's not been that successful...it was to do with how it was done rather than what it was about. I think it's flatlined a little here in our organization. So, what happens is people are designated as trauma-informed champions and various things, and it's wonderful kinds of cosmetic things, and then nothing happens.” Participant 2  Quote 6  “We have this gap and this disparity because staff are saying on the ground… “I need to do this, but I can’t. This isn’t feasible. I am prioritising my wellbeing right now because I am burnt out. And I have so much secondary trauma from working in this service as it is, I can’t even begin to think about changing my practice and saying things differently”…it is not achievable for staff on the ground without the right infrastructure.” Participant 10  Quote 7  “How much capacity does an organization have to work on 50 different projects at the same time?” and ‘how does trauma-informed care get to the top of the list as the most important thing to develop?’ Participant 9  Quote 8  “I mean, there’s the odd reference to it [TI care] in certain policy documents like the substance misuse guidelines and Public Health England have recently mentioned it a few times, but it’s not visible. It is in Scotland and Wales, it’s far more visible and they’ve got policy and legislation, in some cases, to support this, particularly in Scottish Government.” Participant 7  Quote 9  “It comes back down to money…Because if you look at the policies in Scotland, the policies in Wales, and actually in some parts of Ireland, they are allocated…financial resources…which means you can employ staff to do it.” Participant 10  Quote 10  “My sense at the moment, and particularly because COVID has come through and stopped things, it feels like there is a backlog of work and initiatives that have been paused, that are coming from the Department of Health…Actually what the staff need right now is a rest. They need to recover, but actually, there are pressures which are coming.” Participant 9 |
| The evidence-policy gap | Quote 11  “Unfortunately, trauma-informed care, and systemic thinking, hasn’t really infiltrated the way grants are allocated yet. When they say, “There’s not an evidence base for it.” Well, you can’t get the evidence base because you won’t give us the grant to do it.” Participant 3.  Quote 12  “We just don’t have time to actually do this work and then write it up. It is just that you just physically haven’t got that time. You have to prioritise what is important and what isn’t important to get the work done and meet the commission, or meet any deadlines... It is less of a priority- as wrong as this is, to actually spend time writing this up.” Participant 10. |
| The future of TI care in the UK | Quote 13  “I really hope that it gains more traction, meaningful traction, not just nominal traction around, “How many staff have you said you’ve trained, and then you’ve ticked a box and said you’re trauma-informed?” That would be the worst outcome…worse than not doing it at all.” Participant 3 |

Note. TI, trauma-informed.
